# Use of a deep-learning-based lumen extraction method to detect significant stenosis on coronary computed tomography angiography in patients with severe coronary calcification

**DOI:** 10.1186/s43044-022-00280-y

**Published:** 2022-05-21

**Authors:** Hidekazu Inage, Nobuo Tomizawa, Yujiro Otsuka, Chihiro Aoshima, Yuko Kawaguchi, Kazuhisa Takamura, Rie Matsumori, Yuki Kamo, Yui Nozaki, Daigo Takahashi, Ayako Kudo, Makoto Hiki, Yosuke Kogure, Shinichiro Fujimoto, Tohru Minamino, Shigeki Aoki

**Affiliations:** 1 Department of Radiology, Graduate School of Medicine, Juntendo University, 2-1-1 Hongo, Bunkyo-ku, Tokyo 113-8421 Japan; 2 Department of Radiological Technology, Juntendo University Hospital, 3-1-3 Hongo, Bunkyo-ku, Tokyo 113-8421 Japan; 3 Milliman, Inc., Urbannet Kojimachi Bldg, 8F 1-6-2 Kojimachi, Chiyoda-ku, Tokyo 102-0083 Japan; 4 Plusman LLC., 2F 1-3-6 Hirakawacho, Chiyoda-ku, Tokyo 102-0093 Japan; 5 Department of Cardiovascular Biology and Medicine, Graduate School of Medicine, Juntendo University, 2-1-1 Hongo, Bunkyo-ku, Tokyo 113-8421 Japan

**Keywords:** Deep learning, Cycle-GAN, Coronary CT angiography (CCTA), Coronary artery calcification

## Abstract

**Background:**

Coronary computed tomography angiography examinations are increasingly becoming established as a minimally invasive method for diagnosing coronary diseases. However, although various imaging and processing methods have been developed, coronary artery calcification remains a major limitation in the evaluation of the vascular lumen. Subtraction coronary computed tomography angiography (Sub-CCTA) is a method known to be able to reduce the influence of coronary artery calcification and is therefore feasible for improving the diagnosis of significant stenosis in patients with severe calcification. However, Sub-CCTA still involves some problems, such as the increased radiation dose due to plain (mask) imaging, extended breath-holding time, and misregistration due to differences in the imaging phase. Therefore, we considered using artificial intelligence instead of Sub-CCTA to visualize the coronary lumen with high calcification. Given this background, the present study aimed to evaluate the diagnostic performance of a deep learning-based lumen extraction method (DL-LEM) to detect significant stenosis on CCTA in 99 consecutive patients (891 segments) with severe coronary calcification from November 2015 to March 2018. We also estimated the impact of DL-LEM on the medical economics in Japan.

**Results:**

The DL-LEM slightly improved the per-segment diagnostic accuracy from 74.5 to 76.4%, and the area under the curve (AUC) slightly improved from 0.752 to 0.767 (*p* = 0.030). When analyzing the 228 segments that could not be evaluated because of severe calcification on the original CCTA images, the DL-LEM improved the accuracy from 35.5 to 42.5%, and the AUC improved from 0.500 to 0.587 (*p* = 0.00018). As a result, DL-LEM analysis could have avoided invasive coronary angiography in 4/99 cases (per patient). From the calculated results, it was estimated that the number of exams that can be avoided in Japan in one year is approximately 747 for invasive coronary angiography, 219 for fractional flow reserve, and 248 for nuclear exam. The total amount of medical fee that could be reduced was 225,629,368 JPY.

**Conclusions:**

These findings suggest that the DL-LEM may improve the diagnostic performance in detecting significant stenosis in patients with severe coronary calcification. In addition, the results suggest that not a small medical economic effect can be expected.

## Background

Technological advances in imaging equipment and processing have improved image quality and diagnostic ability, and as a result, coronary computed tomography angiography (CCTA) examinations are increasingly becoming established as a minimally invasive method for diagnosing coronary diseases [1–5]. However, although various imaging and processing methods have been developed, coronary artery calcification remains a major limitation in the evaluation of the vascular lumen [6–8].

Subtraction coronary computed tomography angiography (Sub-CCTA) is a method known to be able to reduce the influence of coronary artery calcification and is therefore feasible for improving the diagnosis of significant stenosis in patients with severe calcification. However, Sub-CCTA still involves some problems, such as the increased radiation dose due to plain (mask) imaging, extended breath-holding time, and misregistration due to differences in the imaging phase [9–11]. Therefore, we considered using artificial intelligence (AI) instead of Sub-CCTA to visualize the coronary lumen with high calcification.

Although many CCTA imaging processes utilize artificial intelligence (AI), no algorithm currently exists for visualizing coronary lumen with severe calcification [12–15]. Deep learning-based image processing may solve these problems and improve the diagnostic performance of calcified lesions.

Given this background, the present study aimed to evaluate the diagnostic performance of a deep learning-based lumen extraction method (DL-LEM) to detect significant stenosis on CCTA in patients with severe coronary calcification. We also estimated the impact of DL-LEM on the medical economics in Japan.

## Methods

### Participants

During the period from November 2015 to March 2018, a total of 1797 CCTA were performed. From this cohort, we selected 123 consecutive cases with severe calcification that could not be evaluated in at least one segment and who had undergone invasive coronary angiography (ICA). Among them, 9 cases with poor image quality due to motion artifacts and poor contrast, 5 cases without raw CT data, 1 case for which ICA results could not be confirmed, and 9 cases in which unevaluable segments were placed in the peripheral branch were excluded. Based on the above results, 99 cases were evaluated.

This study was approved by the ethics committee of our institution (H17-0092), and written informed consent was waived due to the retrospective nature of the study. All patients received 0.6 mg sublingual nitroglycerine (Nitropen; Nippon Kayaku, Tokyo, Japan). Patients with a heart rate of ≥ 60 bpm were given 20–40 mg of metoprolol (Lopressor®; Tanabe Seiyaku, Osaka, Japan) orally at 1 h before scanning. If the heart rate remained at ≥ 60 bpm before scanning, 0.125 mg/kg of landiolol (Corebeta®; Ono Pharmaceutical, Osaka, Japan) was administered intravenously.

### CCTA acquisition

All patients underwent CCTA with a 320-row computed tomography (CT) scanner (Aquilion ONE ViSION Edition or Aquilion ONE GENESIS Edition; Canon Medical Systems, Otawara, Japan). CT images were acquired at a tube voltage of 100 kVp or 120 kVp depending on body weight (120 kVp for body mass index [BMI] ≥ 30 kg/m^2^) [16]. The tube current was calculated by automatic exposure control (target standard deviation = 22). For the 320-row CT, the detector collimation was 320 × 0.5 mm and gantry rotation time was 275 ms. The field of view was adjusted to encompass only the heart. The craniocaudal range was between 240 rows (12 cm) and 320 rows (16 cm) to include the entire coronary tree. As the contrast agent, iomeprol (Iomeron® 350 mg I/mL; Eisai, Tokyo, Japan) was injected for 12 s at 18 mg I/kg/s (Dual Shot GX 7; Nemoto Kyorindo, Tokyo, Japan), followed by 30 mL of saline at the same injection rate [17]. Using bolus tracking, scanning was started when the CT attenuation value in the ascending aorta reached 300 Hounsfield units (HU). All patients underwent prospective electrocardiogram-gated CCTA.

### Deep learning model

The DL-LEM [18] used in the present study inputs the unsegmented (un-postprocessed) CCTA dataset and outputs an image with the calcification removed.

### Hardware and software

The model training and validation experiments were performed on a computer with a 64 GB CPU memory, a Xeon E5-2670 v3 CPU (Intel, Santa Clara, CA, USA), and a TITAN Xp graphics processing unit (NVIDIA, Santa Clara, CA, USA), using Chainer 3.2.0, a deep learning framework written in Python 3.6 (http://chainer.org/).

### Input data

Data input to the deep learning model for training were 30 pairs of non-contrast CT images in Digital Imaging and Communications in Medicine (DICOM) format acquired for calcium scoring (reconstruction thickness 2 mm, pitch 2 mm) and the original CCTA axial images (reconstruction thickness 0.5 mm, pitch 0.3 mm) that are routinely sent to a clinical workstation (reconstruction method: AIDR 3D Standard, reconstruction function: FC 04). The training datasets were separated from the patient datasets, and no special segmentation or reconstruction processes were performed before input.

### Details of the deep learning model

**Fig. 1:**
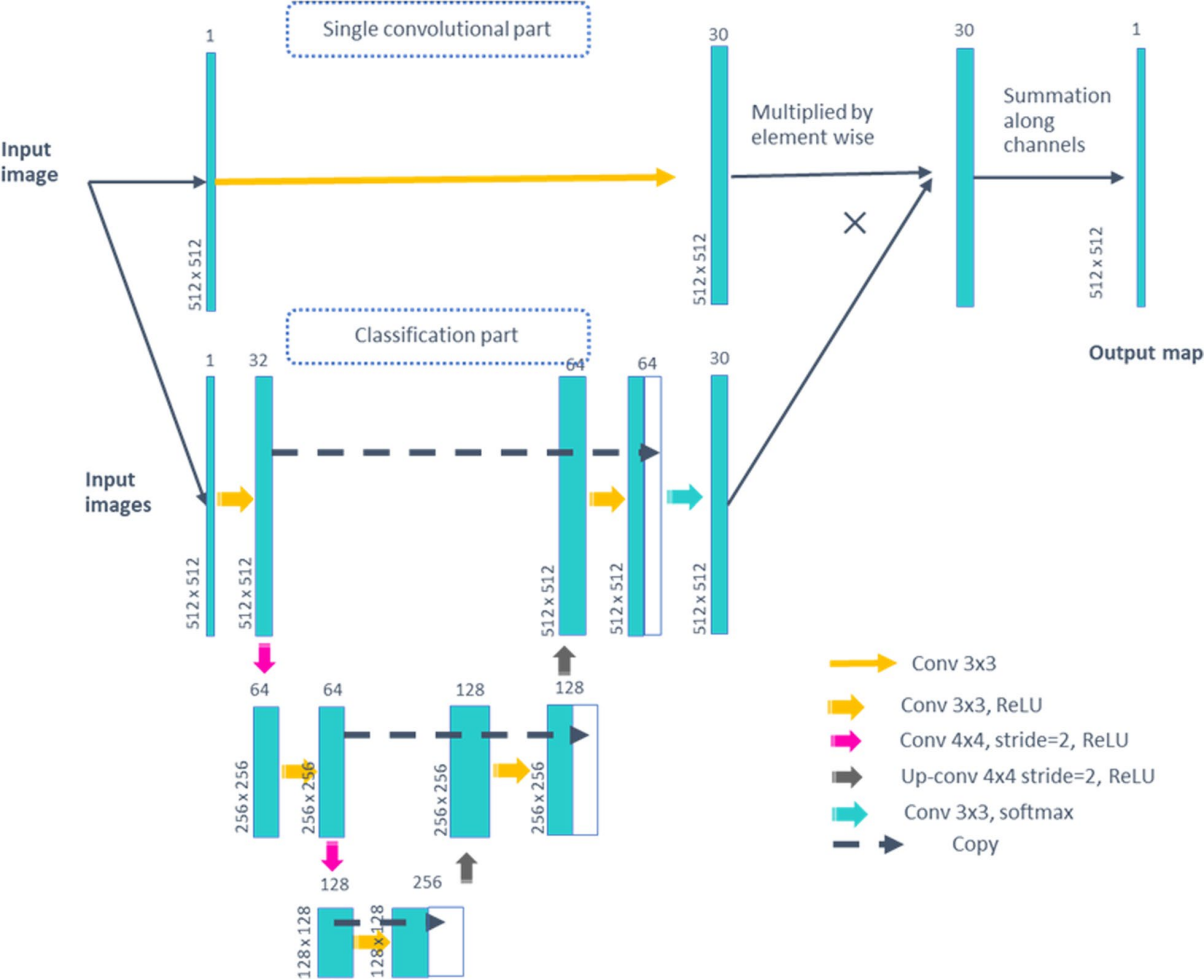
The architecture of 2D mapping functions that translate CCTA and non-contrast CT images. The network is designed to output the weighted average of single convolutions of 3 × 3 (“Single convolutional part”) by pixel-wise classification results by the U-net (“Classification part”). The pixel-wise classification results are softmax-activated so that all classification scores add up to 1. *Conv* convolution, *ReL U* rectified linear unit

The precise architecture of the two-dimensional mapping functions that translate CCTA and non-contrast CT images is described in Fig. 1. Because the process was performed in two dimensions, not three dimensions, the total number of the images used to establish the model was 3000. These two mapping functions (CCTA → non-contrast CT, and vice versa) were trained in the same way as Cycle-Generative Adversarial Network (GAN), which was previously described [18]. We did not assign random noise to the generator model because the objective was simply to learn the mapping of CCTA images into non-contrast CT images. Original DICOM images were cropped from − 1000 to 2000 HU to be input as mapping functions. No other preprocessing was performed. A discriminator was designed to discriminate actual non-contrast CT images from the generated pseudo-non-contrast CT images [19]. In this way, the generative model received information regarding the actual non-contrast CT images and learned to create more realistic pseudo-non-contrast CT images.

**Fig. 2:**
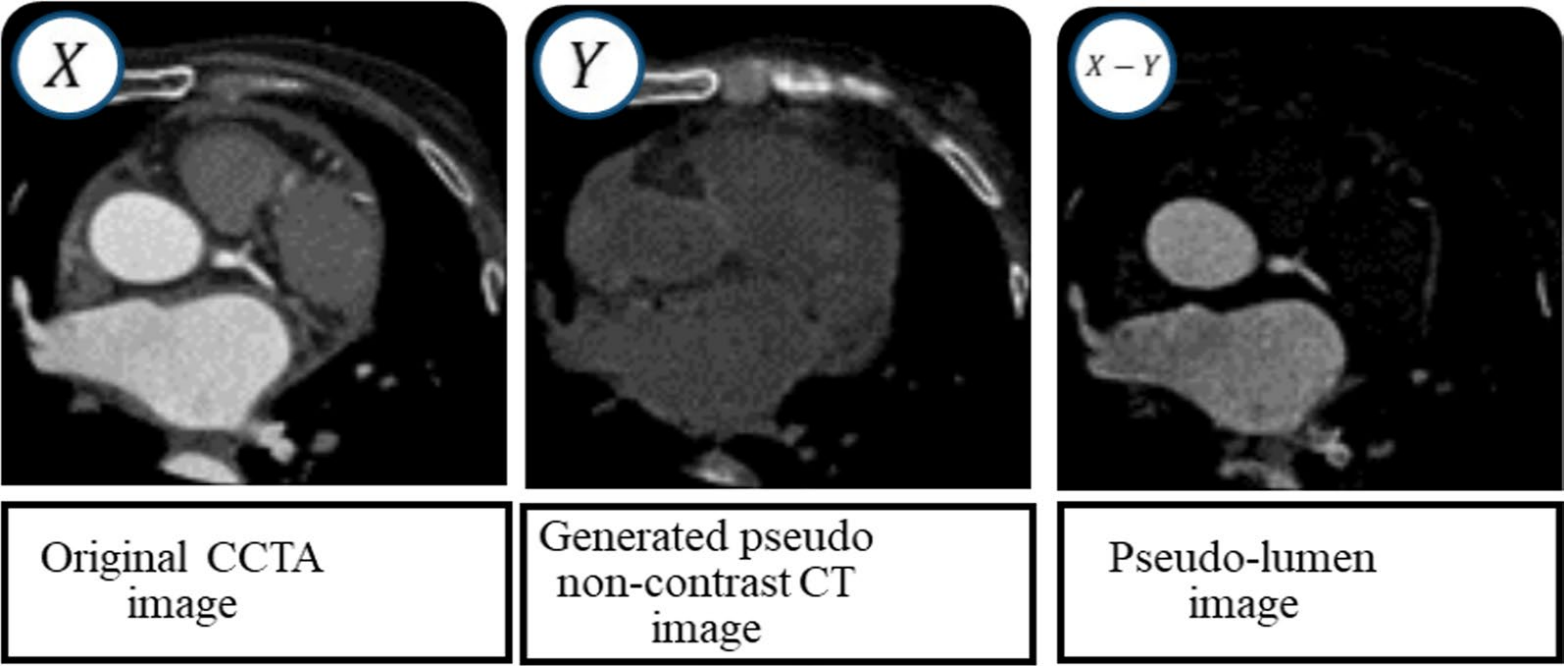
Creation of the pseudo-lumen image

We then subtracted the pseudo-non-contrast CT images from the original CCTA images to create a pseudo-lumen image (Fig. 2). This process was performed slice-by-slice for all CCTA data (DL-LEM images). During this sequence, the generative model learned how to translate CCTA images into non-contrast CT images, i.e., the generative model learned to turn off the contrast enhancement. All data used in the training were unlabeled (CCTA and non-contrast CT). The hyper-parameter settings for the training were as follows: the mini-batch size was 25, Adam [20] alpha (step size) was 0.0002 for both the generator and the discriminator, and Adam beta1 and beta2 (exponential decay rates) were 0.5 and 0.999, respectively.

The time to derive the DL-LEM algorithm was 24 h, and the time to produce the lumen-extracted image for each case was approximately 2 min.

### Stenosis evaluation

Curved planar reconstruction images of the right coronary artery, left anterior descending artery, and left circumflex artery were created on a workstation (Ziostation 2; AMIN, Tokyo, Japan) based on the CCTA and DL-LEM image data. One radiologist and one radiological technologist evaluated the severity of stenosis in the DL-LEM and original CCTA images. The results showed a high concordance rate between the two evaluators. The CCTA and DE-LEM images and ICA were evaluated as having stenosis severities of 0%, 25%, 50%, 75%, 90%, and 100%; segments with ≥ 75% stenosis were considered significant. Next, we compared the diagnostic accuracy for detecting significant stenosis (≥ 75% diameter stenosis) between the two methods with ICA as the reference standard. The evaluation targets were nine segments of the main trunk of three coronary arteries (American Heart Association classification: #1, #2, #3, #5, #6, #7, #8, #11, and #13) [21]. The total number of evaluation segments was 891 (nine segments each from 99 cases).

### Comparison of diagnostic performance for significant stenosis

#### Evaluation 1

**Fig. 3:**
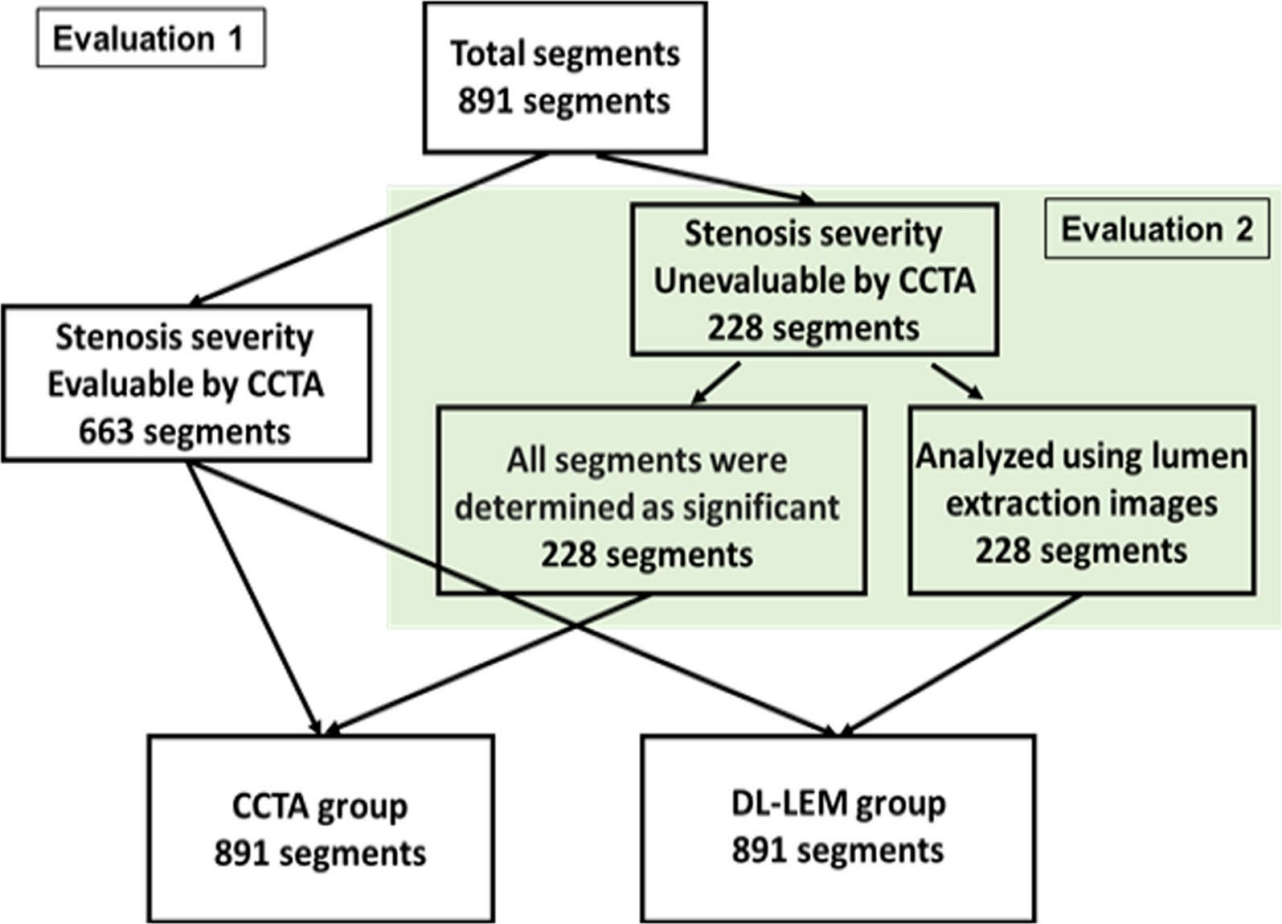
All 891 segments were evaluated in Evaluation 1, while 228 segments with unevaluable segments because of severe calcification were evaluated in Evaluation 2 (shaded area)

All 891 segments were evaluated using either the original CCTA or DL-LEM images and compared with the ICA results. Of the 891 segments evaluated, 228 that were difficult to evaluate by CCTA because of severe calcification were considered significant stenosis (CCTA group). These 228 segments were then analyzed using DL-LEM images (DL-LEM group). The flowchart of this evaluation is shown in Fig. 3.

#### Evaluation 2

We focused on the 228 segments that could not be evaluated by CCTA. These segments were considered significant stenosis in the CCTA group, whereas the DL-LEM images were used in the DL-LEM group. The flowchart of this evaluation is shown in Fig. 3.

### Estimated medical economic effect

Using the total number of CCTA in Japan in 2019 extracted from the 5th NDB Open Data of the Ministry of Health, Labor and Welfare in Japan (https://www.mhlw.go.jp/stf/seisakunitsuite/bunya/0000177221_00008.html), we estimated the number of unevaluable segments due to calcification. We estimated the number of downstream ICA, FFR, and nuclear exam based on the CCTA database of our hospital during the study period. Based on the results of this study, we calculated the number of downstream exams that could be canceled and also evaluated the total medical cost that could be saved. The fee for ICA, FFR, and nuclear exam were 200,000 Japanese Yen (JPY), 440,000 JPY, and 95,441 JPY, respectively.

### Statistical analysis

Statistical analysis was carried out using R version 4.0.2 (R Foundation for Statistical Computing) [22, 23]. The diagnostic performance, including sensitivity, specificity, accuracy, positive predictive value, and negative predictive value, of the CCTA and DL-LEM groups in identifying ≥ 75% stenosis severity was evaluated. The accuracy was tested using McNemar’s test, and the other four evaluation items were tested using Fisher’s exact test. Receiver operating characteristic (ROC) analysis was used to test the accuracy of the CCTA and DL-LEM groups as the reference method in identifying coronary significant stenosis. Values of *p* < 0.05 were considered to indicate a significant difference. An area under curve (AUC) of 0.50 was considered a valueless diagnosis, 0.50 < AUC ≤ 0.7 low diagnostic accuracy, 0.7 < AUC ≤ 0.9 moderate diagnostic accuracy, and 0.9 < AUC < 1.0 good diagnostic accuracy [12]. In addition, net reclassification improvement (NRI) and integrated discrimination improvement (IDI) were calculated, with *p* < 0.05 considered to indicate a significant difference.

## Results

The characteristics of the patient population are summarized in Table 1. The gender ratio (male/female) was 75:24 and the patients age ranged from a minimum of 40 years to a maximum of 95 years, with a median of 75 years. The average Agatston score was 902.7, and more than half of the patients had hypertension, hyperlipidemia, or diabetes mellitus as an underlying disease. Since the average BMI was 24.0 kg/m^2^, the imaging tube voltage was often 100 kVp.

**Table 1:** Characteristics of the patients in this study

Gender (M/F)	75/24
Age (years, mean ± SD)	73.1 ± 9.7
Height (cm, mean ± SD)	162.7 ± 9.2
Weight (kg, mean ± SD)	64.0 ± 12.1
Body mass index (kg/m^2^, mean ± SD)	24.0 ± 3.2
Family history	32.3%
Hypertension	67.7%
Hyperlipidemia	63.6%
Diabetes mellitus	51.5%
Smoking	18.2%
Agatston score (total, mean ± SD)	902.7 ± 499.5
Tube voltage 100/120 (kVp)	76/23

*SD* standard deviation, *kVp* kilovolt peak

The concordance rate of the stenosis severity evaluations of the two evaluators was 86.8%.

### Evaluation 1

**Fig. 4:**
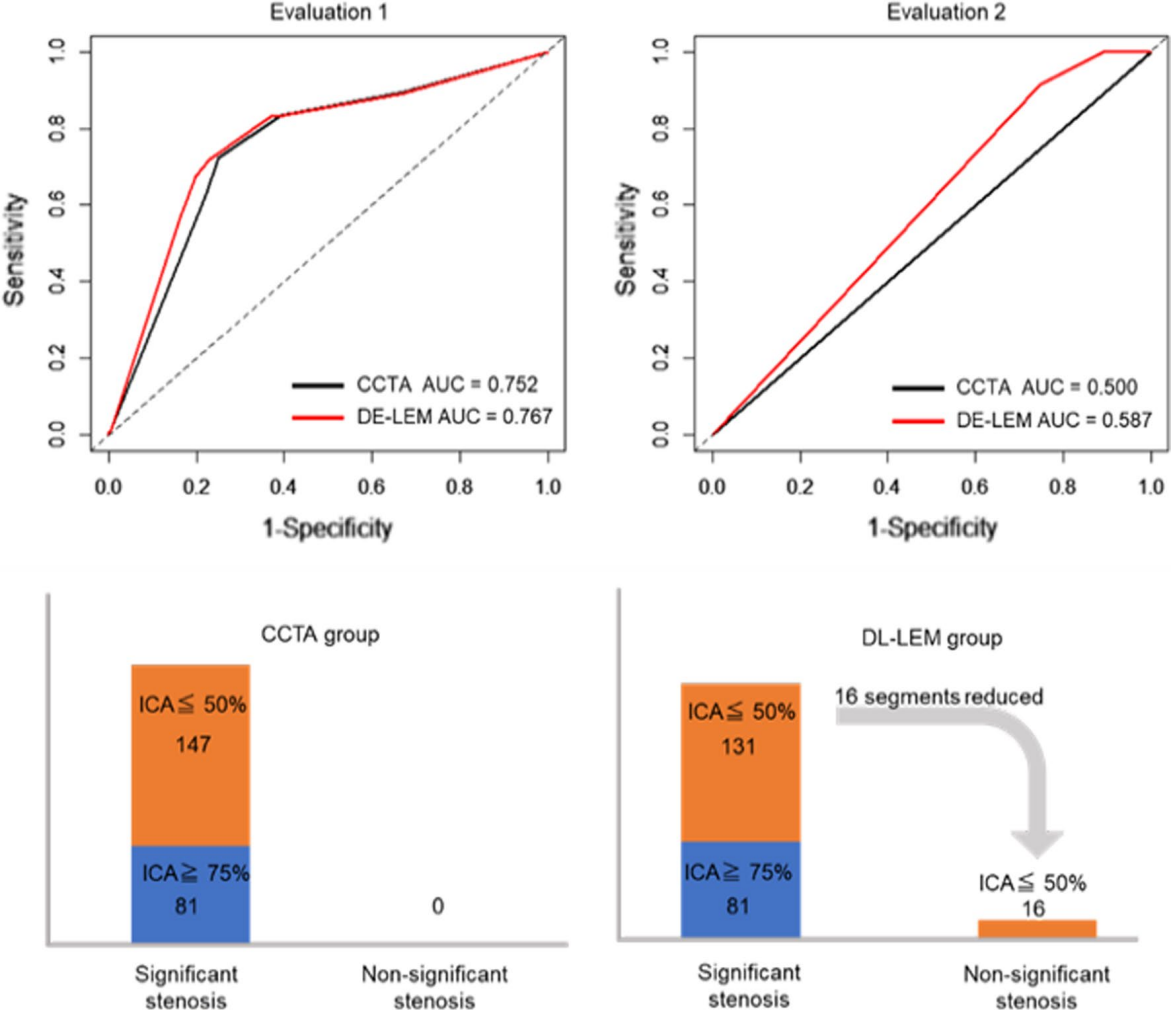
The DL-LEM significantly improved the AUC of the ROC curve (Evaluation 1: *p* = 0.030, Evaluation 2: *p* < 0.001). The DL-LEM could reduce 16 of 147 segments by identifying false positives

**Fig. 5:**
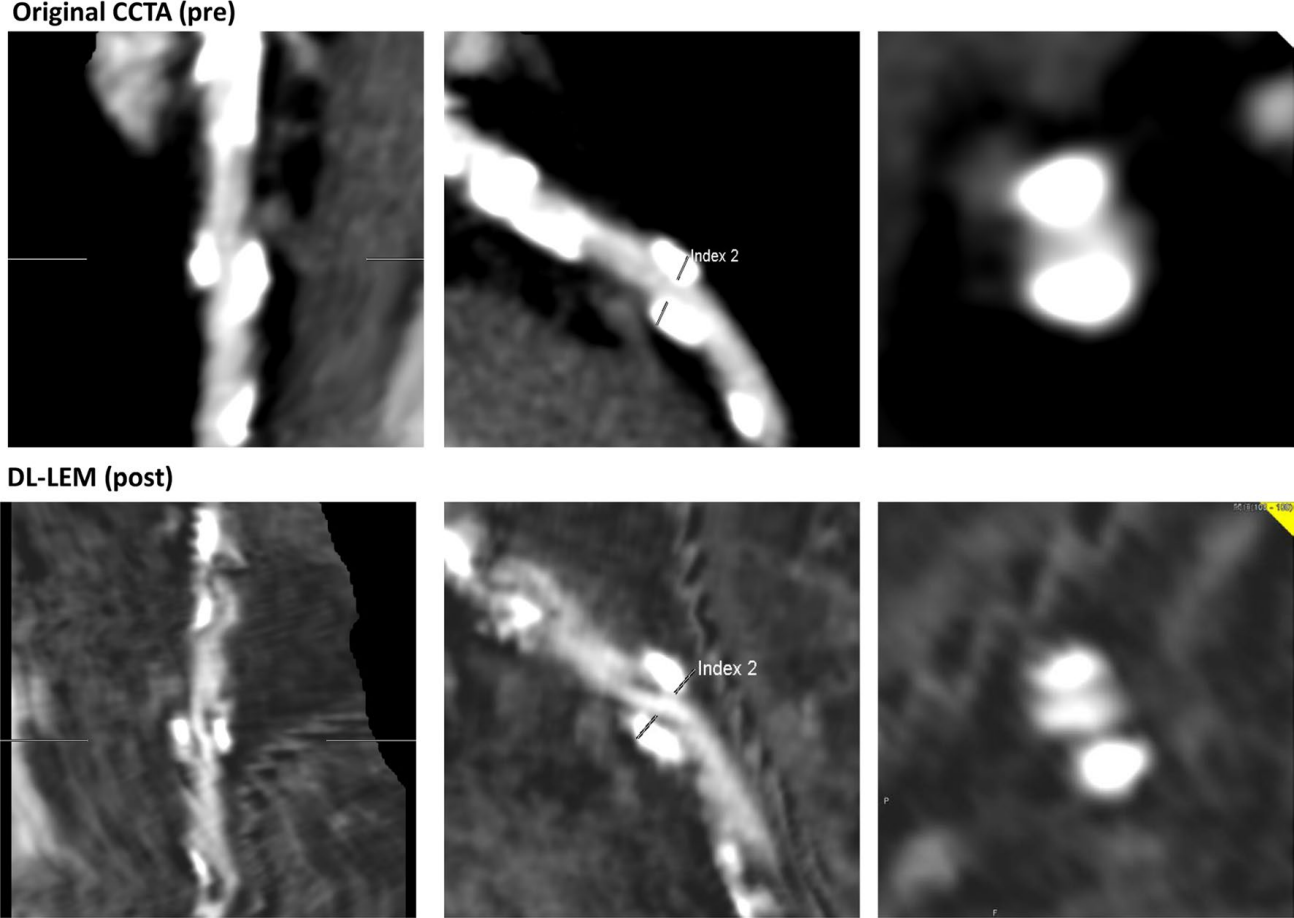
The LAD (#8) in a 77-year-old male patient was determined as showing significant stenosis because of severe calcification by using original CCTA data. The DL-LEM reduced the calcification volume and could provide a diagnosis of nonsignificant stenosis

Of the 303 segments considered to be significant stenosis by CCTA (including 228 segments that were unevaluable due to calcification), 179 were diagnosed as nonsignificant stenosis by ICA, but DL-LEM reduced the number of false positives by 16 segments (Table 2). With ICA as the reference standard for identifying ≥ 75% diameter stenosis, the sensitivity and specificity were slightly improved (from 72.1 to 72.7% and from 75.1 to 77.3%, respectively) by using DL-LEM, but these differences were not significant. By contrast, the diagnostic accuracy was significantly improved (from 74.5 to 76.4%; *p* < 0.001) (Table 3). The AUC was significantly higher in the DL-LEM compared with the CCTA group (AUC_DL-LEM_ = 0.767, 95% confidence interval [CI]: 0.727–0.808, AUC_CCTA_ = 0.752, 95% CI: 0.713–0.791; *p* = 0.030) (Fig. 4). NRI and IDI were also significantly different between the CCTA and DL-LEM groups (NRI = 0.457, 95% CI: 0.328–0.586, *p* = 0.000, IDI = 0.020, 95% CI: 0.012–0.027, *p* = 0.000). As shown in Fig. 5, DL-LEM analysis could have avoided ICA in 4/99 cases (per patient) and 16/228 cases (per segment).

**Table 2:** Comparison of CCTA and DL-LEM in detecting significant stenosis (≥ 75%) with ICA as the reference standard

	CCTA group		DL-LEM group	
	Significant stenosis (≥ 75%)	Nonsignificant stenosis	Significant stenosis (≥ 75%)	Nonsignificant stenosis
*Evaluation 1*				
ICA significant stenosis (≥ 75%)	124	48	125	47
ICA nonsignificant stenosis	179	540	163	556
*Evaluation 2*				
ICA significant stenosis (≥ 75%)	81	0	81	0
ICA nonsignificant stenosis	147	0	131	16

*CCTA* coronary computed tomography angiography, *DL-LEM* deep learning-based lumen extraction method, *ICA* invasive coronary angiography

**Table 3:** The diagnostic performance of CCTA and DL-LEM in identifying ≥ 75% stenosis

	Evaluation 1		Evaluation 2	
	CCTA group	DL-LEM group	CCTA group	DL-LEM group
Sensitivity	72.1% (64.8–78.7%)	72.7% (65.4–79.2%)	100% (93.4–100%)	100% (93.4–100%)
Specificity	75.1% (71.8–78.2%)	77.3% (74.1–80.3%)	0% (0–3.7%)	10.9%** (6.4–17.1%)
Positive predictive value	40.9% (35.3–46.7%)	43.4% (37.6–49.3%)	35.5% (29.3–42.1%)	38.2% (31.6–45.1%)
Negative predictive value	91.8% (89.3–93.9%)	92.2% (89.8–94.2%)	N/A (0–100%)	100% (71.3–100%)
Accuracy	74.5% (71.5–77.4%)	76.4%^**^ (73.5–79.2%)	35.5% (29.3–42.1%)	42.5%^**^ (36.0–49.2%)

*N/A* not available

**p < 0.01. Data are presented with 95% confidence intervals in parentheses

As an additional downstream testing, 41 patients underwent nuclear exam, although the 4 cases which could be ruled-out by DL-LEM was not included. Nuclear exam could rule out 27 patients (66%) and diagnose 1 patient (2%) correctly for having significant stenosis by ICA, but there were 9 patients (22%) with false positives and 4 patients (10%) with false negatives.

### Evaluation 2

With ICA as the reference standard for identifying ≥ 75% diameter stenosis, the DL-LEM significantly improved the specificity from 0 to 10.9% (*p* < 0.001) and the diagnostic accuracy from 35.5 to 42.5% (*p* < 0.001) (Table 3). The AUC was significantly higher in the DL-LEM than in the CCTA group (AUC_DL-LEM_ = 0.587, 95% CI: 0.542–0.633; AUC_CCTA_ = 0.500, 95% CI: 0.500–0.500; *p* < 0.001) (Fig. 4). NRI and IDI were significantly different between the CCTA and DL-LEM groups (NRI = 0.331, 95% CI: 0.144–0.517, *p* = 0.0005; IDI = 0.0525, 95% CI: 0.028–0.077, *p* < 0.001).

### Estimated medical economic effect

The number of CCTA cases at our hospital during the study period was 1,797, and 235 cases (13.1%) could not be evaluated due to severe calcification even in one segment. Of the 235 cases, 123 cases underwent ICA (52.3%) and 36 cases also underwent FFR measurement (29.3% of total ICA cases). In addition, 41 cases underwent nuclear exam instead of ICA (17.4%). The ICA avoidance rate using DL-LEM was 4.04% (4 out of 99 cases) in this study.

According to the 5th NDB Open Data of the Ministry of Health, Labor and Welfare in Japan, the total number of CCTA cases conducted in Japan in 2019 was 270,252. Based on the data in the present study, 35,342 cases (13.1%) would not be evaluated in at least one segment due to calcification. Of these, ICA would be performed in 18,484 cases (52.3%), of which 5,416 cases (29.3%) would undergo FFR evaluation, and nuclear exam would be performed in 6,149 cases (17.4%). Of the 18,484 cases in which ICA is performed, DL-LEM could avoid 747 cases, including 219 FFR evaluations. In addition, nuclear exam could be avoided in 248 cases. Therefore, the total medical cost savings would be 225,629,368 JPY (105,600,000 JPY for ICA reduction, 96,360,000 JPY for FFR reduction, and 23,629,368 JPY for nuclear exam reduction, respectively).

## Discussion

CCTA is considered to have good diagnostic ability for coronary stenosis, but severe calcification remains a limitation. In this study, we examined whether the DL-LEM, which removes the calcified region of the coronary artery to visualize the coronary lumen, could improve diagnostic ability in cases of severe calcification. The results indicated that the DL-LEM could significantly improve diagnostic ability in both evaluations. The DL-LEM also reduced 16 of 228 segments by identifying false positives, which could not be identified by CCTA, meaning that 4.0% of patients could have avoided ICA. In Japan, inexpensive modalities such as stress echo or stress electrocardiogram is not recommended as additional exam when coronary stenosis is suspected at CCTA [24]. Nuclear exam is recommended, but in this study approximately 30% of the patients showed either false positive or false negative. Special care might be necessary when interpreting nuclear exam as an additional testing in patients with severe calcification.

Although the clinical improvement using DL-LEM might be small, it was estimated that 731 ICAs, 212 FFRs, and 239 RIs could be reduced as a nation. The amount of medical fee reduction was approximately 220 M JPY. These results shows that the impact of DL-LEM on medical costs is not small.

Although it has been a long-standing problem to diagnose severe coronary artery stenosis with severe calcification using a minimally invasive method, to our knowledge, no previous study has attempted to visualize the lumen of coronary arteries with severe calcification using deep learning. Among the various methods have been investigated, fractional flow reserve CT (FFR_CT_) has been shown to be more diagnostically accurate than other modalities (e.g., CCTA, single-photon emission CT, positron emission tomography). In addition, the combination of CCTA and FFR_CT_ has been reported to be useful in determining treatment strategies for patients with noninvasive coronary artery disease [25]. FFR_CT_ has been reported to achieve high diagnostic performance even in patients with coronary artery calcification, but the diagnostic accuracy of FFR_CT_ may be low in patients with severe coronary calcification (Agatston score > 1000) [26]. On the other hand, in the present study, the DL-LEM improved the diagnostic ability, even in patients with an Agatston score close to 1000 (mean 902.7 ± 499.5).

Guidelines might be against performing CCTA in patients with severe coronary calcification (i.e., Agatston score > 1000). In our institution, we do not have a particular threshold to cancel CCTA due to heavy calcification at the time of the scan. This is because even when coronary angiography is performed, knowledge of coronary anatomy as well as the severity of calcification would support interventional cardiologists in guiding the intervention procedure. The results of this study would benefit patients who undergo CCTA in spite of severe calcification.

CT perfusion (CTP) is a new modality that improves the sensitivity and specificity of CCTA that has already been integrated into clinical practice for the evaluation of patients with suspected coronary artery disease. A previous study of patients with severe coronary artery calcification (Agatston score > 400) reported improved evaluation of coronary artery lesions [27]. Adding CTP to CCTA may therefore yield more reliable results for cases of severe calcified lesions that were previously difficult to diagnose. However, although recent technological advances have led to improvements in CTP, it still involves some limitations, such as complicated imaging protocols and high radiation doses. Compared with CTP, the DL-LEM performs diagnoses using images retrospectively created from CCTA images, so the exposure dose is the same as that in normal CCTA.

Sub-CCTA is a technique that removes calcification of the coronary arteries by subtracting a pre-scan from a contrast enhancement scan. This method of removing the calcification and evaluating the stenosis of the coronary arteries is the same as the output of the DL-LEM examined in this study. Previous studies have reported that the proportion of image quality segments that cannot be diagnosed because of coronary artery calcification is 43.9% in conventional CT, but can be improved to 8.5% by using Sub-CCTA [10]. Although Sub-CCTA is considered to be useful as a method for diagnosing calcified coronary arteries morphologically, it still has some problems. First, pre- and post-scans can cause misregistration artifacts. According to a previous study [10], the breath-holding time for two scans is 20–40 s; thus, there is a risk of misregistration due to poor breath-holding and fluctuations in heart rate. Second, the exposure dose is almost twice that of normal CCTA because of the need for pre- and post-scans. The average estimated effective radiation dose for Sub-CCTA, which is the sum of the pre-contrast and contrast-enhanced scans, is reportedly 3.20 mSv (range, 1.16–8.80 mSv). On the other hand, since the DL-LEM examined in this study uses normal CCTA images, the breath-holding time was within 10 s. In addition, the average estimated effective dose in this study was lower than that for Sub-CCTA, at 2.77 mSv (range, 1.07–8.13 mSv). These points are considered to be advantages of the DL-LEM compared with Sub-CCTA.

The analysis using the DL-LEM examined in this study was performed using complete post-processing, and the coronary arteries were not manually segmented. The coronary arteries are automatically recognized using deep learning, and this is considered a strength; however, calcification cannot be completely removed in all parts because of the accuracy of the DL-LEM. Whether or not to perform downstream testing when the stenosis grade was initially severe at CCTA but was downgraded to mild to moderate using the DL-LEM depends on the pretest probability. If the patient has no or minimal symptoms, we would not recommend further testing. However, if typical or atypical chest pain is present, we would suggest additional stress test, because some lesions with mild stenosis might cause ischemia.

This study did have some limitations. First, as mentioned above, it was not possible to remove the calcification completely because of the accuracy of the DL-LEM. Therefore, for severely calcified segments, the improvement in diagnostic ability is limited owing to the underestimation of the coronary artery lumen. Therefore, improving the accuracy of calcification removal is considered to be a future task for the DL-LEM. In addition, this study was performed in a single facility using a single CT device and workstation; therefore, the results may differ when analysis is carried out under different conditions. Second, we did not investigate the effect of different types of calcification, such as intimal or medial artery calcification, on the performance of DL-LEM. Further study is necessary to uncover this issue. Third, we did not investigate the relationship between the efficacy of DL-LEM and the severity of coronary calcification due to the limited number of cases, which could be investigated in a study including a large number of cases.

## Conclusions

The DL-LEM appears to be useful for the diagnosis of severely calcified coronary artery lesions. DL-LEM would also have a nonignorable impact on the medical economics. If a segment cannot be diagnosed because of severe calcification in daily coronary CT examinations, it can be diagnosed by subtracting calcification with post-processing. Unfortunately, the accuracy of deep learning models is not yet sufficient, so the effects are still limited. However, anticipating future progress, the DL-LEM can be expected to be used as software that can be handled on-site on the console of CT devices.

## Data Availability

The data that support the findings of this study are available on reasonable request from the corresponding author [NT]. The data are not publicly available because they contain information that could compromise research participant privacy/consent.

## Abbreviations

DL-LEM: Deep learning-based lumen extraction method
CCTA: Coronary computed tomography angiography
CT: Computed tomography
ICA: Invasive coronary angiography
AUC: Area under the curve
FFR: Fractional flow reserve
Sub-CCTA: Subtraction coronary computed tomography angiography
AI: Artificial intelligence
HU: Hounsfield units
DICOM: Digital imaging and communications in medicine
GAN: Generative adversarial network
JPY: Japanese yen
ROC: Receiver operating characteristic
NRI: Net reclassification improvement
IDI: Integrated discrimination improvement
BMI: Body mass index
kVp: Kilovolt peak
CTP: Computed tomography perfusion
